# Supplementary material for: Vibrational noise disrupts Nezara viridula communication, irrespective of spectral overlap
Source: Commun Biol. 2024 Nov 19;7:1533. doi: 10.1038/s42003-024-07185-3 (PMC11577028; doi:10.1038/s42003-024-07185-3)
Supplement: Supplementary file 2 — Supplementary information [file 42003_2024_7185_MOESM2_ESM.pdf]

Vibrational noise disrupts *Nezara viridula* communication, irrespective of frequency overlap

Rok Janža, Nataša Stritih-Peljhan, Aleš Škorjanc, Jernej Polajnar, and Meta Virant-Doberlet

GLOSSARY

Table S1: Glossary of less common terms used in the present study

**Definition**	**Reference**
Fundamental frequency: the principal frequency component of a signal. Also known as the first harmonic frequency.	Howard & Angus (2009)
Higher harmonic frequency: a frequency component of a signal that is a positive integer multiple of the fundamental frequency.	Howard & Angus (2009)
Noise: stimulus not associated with signals of interest to a receiver which can mask the signal features or distract/mislead the receiver.	Classen-Rodriguez et al. (2021), Raboin & Elias (2019)
Masking: consequence of noise which diminishes the receiver's ability to detect a signal or discriminate between signals.	Brumm & Slabbekoorn (2005), Yost (2008), Kidd et al. (2008)
FCS: female calling song, a type of vibrational signal emitted in regular sequences by stationary *Nezara viridula* females during courtship.	Čokl et al. (2000)
FON: fundamental frequency-overlapping noise. Noise stimulus spanning the 50-150 Hz band, designed to overlap the FCS fundamental frequency.	*this study*
FHON: Fundamental and harmonic-overlapping noise. Noise stimulus spanning the 50-500 Hz band, designed to overlap the FCS fundamental frequency and higher harmonics.	*this study*
NON: non-overlapping noise. Noise stimulus spanning the 500-1000 Hz band, above the FCS frequencies.	*this study*
Vibroscape: A collection of biological, geophysical and anthropogenic vibrations emanating from a given landscape to create unique vibrational patterns across a variety of spatial and temporal scales.	Šturm et al. (2019)
Acoustic/audition: Pertaining to sound; i.e., longitudinal waves in homogenous fluid media.	Hill & Wessel (2016)
Vibrational/vibratory: Pertaining to substrate-borne waves at the boundary between media, with particles oscillating perpendicular to the plane of wave propagation.	Hill & Wessel (2016)
Summed neuronal activity/response: Combined activity of multiple neurons recorded as a single compound signal, typically from nerves containing multiple axons	Strauβ et al. (2019)

SUPPLEMENTARY METHODS

Behavioural assays

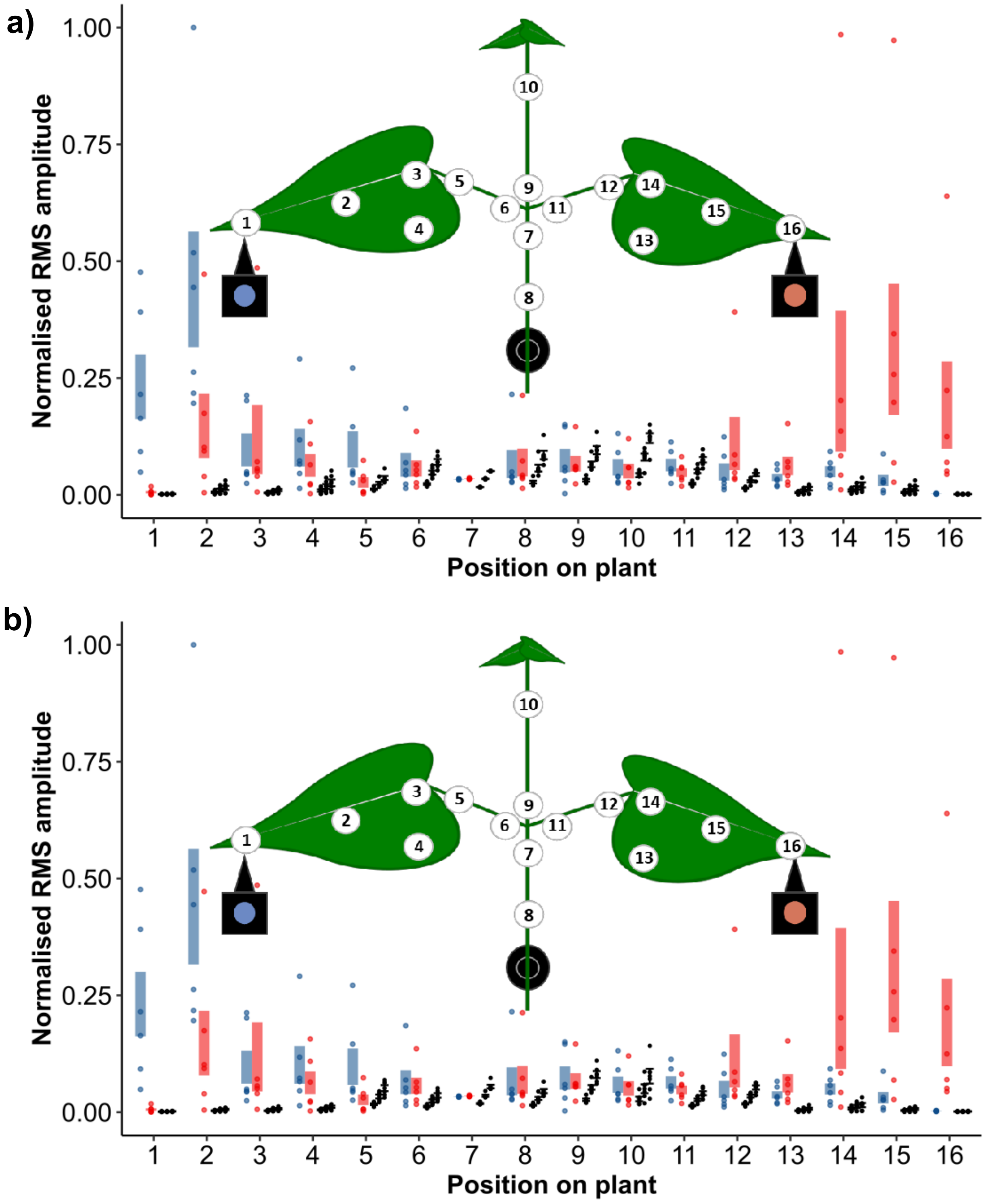

Figure S1: Amplitude levels of FCS, FHON (a) and NON (b) amplitudes registered at different positions on the plant. Fig. 8 in the main text shows the same for FON. The blue and red bars represent means with standard errors for normalised amplitude of FCS played from the minishaker marked with the same colour. The black bars at each position represent means with standard errors of three different amplitude levels of noise: from left to right, 6, 0 and -6 dB SNR relative to FCS measured at the reference point, i.e. point 7.

Table S2: Plant section lengths.

Section	Plant 1	Plant 2	Plant 3	Plant 4	Plant 5	Plant 6
A	100	107	110	124	107	110
B	32	46	46	42	42	47
C	42	45	45	43	41	41
D	105	86	100	92	86	86
E	105	95	100	105	90	82
F	210	175	180	160	175	203

Table S3: RMS amplitudes of vibrations measured on different sections of the plants used in the experiment.

Plant	Position	FCS LS	FCS RS	FON 6 dB	FON 0 dB	FON -6 dB	FHON 6 dB	FHON 0 dB	FHON -6dB	NON 6 dB	NON 0 dB	NON -6 dB
1	1	202	209	117	195	300	95	215	302	100	189	306
1	2	1291	1278	350	666	897	243	557	770	74	128	207
1	3	680	112	270	420	632	184	395	586	61	104	164
1	4	497	515	90	136	202	85	187	269	72	134	217
1	5	23	265	2	2	2	2	2	2	2	2	2
1	6	159	163	4	5	7	5	8	10	4	5	6
1	7	202	167	6	8	12	5	9	12	6	8	12
1	8	558	820	29	46	66	20	44	61	11	13	18
1	9	903	142	374	638	940	253	558	810	121	251	364
1	10	169	93	91	128	210	144	279	450	140	263	427
1	11	1112	156	248	415	630	176	379	558	105	207	308
1	12	1630	15	71	118	177	64	145	194	136	270	414
1	13	2350	1	1	1	1	1	1	1	1	1	1
1	14	1307	29	6	11	15	8	14	20	5	9	10
1	15	1278	37	11	19	29	9	20	28	5	11	15
1	16	1747	16	17	27	40	11	24	33	9	15	22
2	1	195	221	102	206	303	100	202	305	104	198	303
2	2	295	436	189	374	545	148	303	454	86	164	256
2	3	556	490	180	363	535	128	274	397	79	146	229
2	4	746	392	83	150	217	86	178	269	121	230	351
2	5	17	306	3	5	7	3	5	8	3	4	6
2	6	28	413	11	17	22	9	16	22	4	5	6
2	7	123	125	7	13	19	6	10	15	4	6	8
2	8	344	66	25	44	64	15	26	38	12	14	17
2	9	876	367	263	516	751	172	348	525	225	452	663
2	10	544	3552	311	557	788	250	505	755	190	350	519

Plant	Position	FCS LS	FCS RS	FON 6 dB	FON 0 dB	FON -6 dB	FHON 6 dB	FHON 0 dB	FHON -6dB	NON 6 dB	NON 0 dB	NON -6 dB
2	11	313	816	284	563	827	165	344	499	89	179	252
2	12	260	443	145	276	427	114	235	341	90	164	266
2	13	293	8	9	17	24	7	14	20	5	9	14
2	14	2667	1048	14	21	30	8	15	23	5	8	10
2	15	296	307	9	16	24	7	13	18	7	14	20
2	16	84	144	29	55	82	20	40	57	11	16	30
3	1	203	200	101	204	317	105	200	306	106	210	300
3	2	236	229	178	336	506	166	315	480	95	183	272
3	3	300	193	172	354	520	131	298	444	108	207	308
3	4	103	200	64	132	196	90	168	253	131	251	379
3	5	11	1342	7	10	10	8	12	17	8	9	9
3	6	157	2070	46	49	69	35	52	78	29	59	63
3	7	107	245	50	88	134	43	76	110	23	45	57
3	8	251	1213	64	106	152	85	153	223	87	138	191
3	9	356	360	224	435	625	201	384	570	153	296	450
3	10	232	351	268	588	790	298	553	813	183	351	512
3	11	139	260	153	314	475	133	254	382	49	92	137
3	12	309	40	68	135	192	74	141	208	67	128	188
3	13	984	41	7	9	11	7	9	14	7	10	14
3	14	1177	611	48	100	133	54	91	133	31	48	58
3	15	263	426	35	61	80	37	63	93	25	37	53
3	16	437	941	101	195	272	84	169	242	41	69	98
4	1	201	201	89	202	344	96	196	300	121	205	305
4	2	160	210	110	250	419	121	247	383	123	267	392
4	3	148	357	98	211	364	108	216	340	73	153	226
4	4	198	2350	26	50	85	58	119	172	115	219	328
4	5	20	3838	9	9	8	9	9	9	13	10	14
4	6	192	5840	21	41	68	36	63	89	35	52	74
4	7	324	916	26	52	88	30	58	88	23	32	46
4	8	412	5915	50	60	101	40	67	91	58	76	97
4	9	17	877	127	258	452	148	325	449	127	269	394
4	10	239	161	193	411	710	236	466	705	102	208	295
4	11	255	318	99	202	340	105	211	315	52	110	157
4	12	876	184	32	68	112	70	143	210	119	256	379
4	13	2862	108	8	10	10	8	6	7	5	7	8
4	14	6005	2836	28	61	99	31	53	84	22	42	56
4	15	1212	2917	23	43	72	27	51	76	18	36	52
4	16	275	387	47	94	152	64	118	173	45	76	101
5	1	207	207	90	196	297	102	214	312	102	196	322
5	2	229	254	111	220	351	160	327	497	86	167	272
5	3	337	223	126	291	416	144	301	435	103	203	328

Plant	Position	FCS LS	FCS RS	FON 6 dB	FON 0 dB	FON -6 dB	FHON 6 dB	FHON 0 dB	FHON -6dB	NON 6 dB	NON 0 dB	NON -6 dB
5	4	151	219	54	123	166	108	220	316	118	235	376
5	5	8	750	9	9	9	8	9	10	10	9	11
5	6	527	1548	42	82	192	42	82	122	27	31	42
5	7	397	426	41	81	118	45	86	125	22	32	50
5	8	90	254	62	87	111	86	102	140	64	115	109
5	9	308	324	141	325	481	156	328	483	172	335	545
5	10	789	722	208	471	712	273	510	750	297	521	853
5	11	414	387	117	270	369	122	254	368	86	174	273
5	12	281	239	40	93	132	65	131	191	122	244	383
5	13	1291	8	13	13	13	9	10	13	9	10	10
5	14	1577	564	43	78	132	65	125	186	24	38	52
5	15	270	338	26	58	75	34	52	73	23	55	48
5	16	707	654	111	248	353	109	221	312	34	53	77
6	1	194	206	102	216	305	101	211	312	137	273	441
6	2	171	85	171	380	526	152	315	492	78	156	226
6	3	272	342	199	427	565	162	340	509	74	142	210
6	4	67	285	65	137	181	95	186	289	89	172	257
6	5	14	419	15	19	33	21	15	17	14	16	18
6	6	58	1189	68	139	194	62	123	187	34	47	55
6	7	185	355	56	113	176	48	91	134	37	60	94
6	8	135	503	106	200	265	93	150	219	57	83	115
6	9	182	332	242	531	733	207	412	616	161	310	489
6	10	151	156	363	667	936	321	600	902	110	115	173
6	11	85	102	171	360	499	133	274	423	59	113	169
6	12	155	38	48	94	133	52	103	161	78	150	224
6	13	556	41	13	12	17	16	20	20	16	12	18
6	14	3112	237	44	98	148	38	75	95	28	25	30
6	15	148	240	33	62	84	42	59	85	33	52	76
6	16	395	129	54	82	116	48	74	109	42	67	90

Table S4: Parameters of natural FCS (n=16) used for artificial FCS synthesis.

Female	Dominant frequency [Hz]	Signal duration [s]	Pause duration [s]
1	80	0,875	2,6
2	90	NA	NA
3	85	NA	NA
4	82	1	3
5	92	NA	NA
6	92	1	3
7	90	1	3
8	94	1	2,8
9	95	0,8	3
10	80	1,1	NA
11	82	1,2	2
12	90	1	2,5
13	96	1,2	3,8
14	93	1,2	2,6
15	94	1,1	2,7
16	95	1	3
Average	89,4	1,0	2,8

Table S5: RMS amplitudes of *N. viridula* FCS, measured at the reference point below the branching point of the stem and leaves of a representative bean plant. Live females were recorded signalling from a leaf close to the vibration exciter on the setup for behavioural experiments. A calibrated data acquisition device (Sinus Soundbook mk2 with Samurai 3.0 software, Sinus Messtechnik GmbH, Germany) was used to calculate absolute amplitudes.

Female	RMS amplitude [mm/s]
1	0.147
2	0.600
3	0.698
4	0.548
5	1.890
6	0.207
7	0.158
8	1.193

Electrophysiological recordings from leg nerves

Stimuli synthesised in Matlab and Audacity as described in the main text were imported into Spike2 software (Cambridge Electronic Design, UK), which enabled both stimulation and registration of extracellularly recorded nerve impulses, i.e. spikes, via the CED Power 1401 interface (Cambridge Electronic Design, UK). We fed the stimuli through the DAC channel to a Rigol PA1011 power amplifier and a custom-built attenuator prior to reaching the vibration exciter.

The measuring electrode, consisting of an $Ag/AgCl_2$ wire inserted into a borosilicate glass capillary (Sutter Instruments, USA) filled with Davenport saline, was prepared using a P-97 micropipette puller (Sutter Instruments, USA). The capillary was fixed in a holder connected to a suction attachment (Gilmont Instruments, Inc., USA) allowing to establish a sealed contact between the nerve and the electrode. The electrode's signal was amplified with the ISO-80 amplifier (WPI, USA) and passed a HumBug Noise Eliminator (Digitimer, USA) to reduce electronic noise. The processed signal was sent to the CED Power 1401 interface (20 kHz sample rate) for recording to a PC via Spike 2 software. Spike detection and sorting were performed using threshold detection and template matching methods in Spike 2, with inadequate spikes excluded from the analysis. In some recordings distinct spikes of individual sensory cells were observed, while in most cases, the activity represented a summed response from undistinguishable units. Timestamps of individual spikes were exported either to Matlab or R for further analysis.

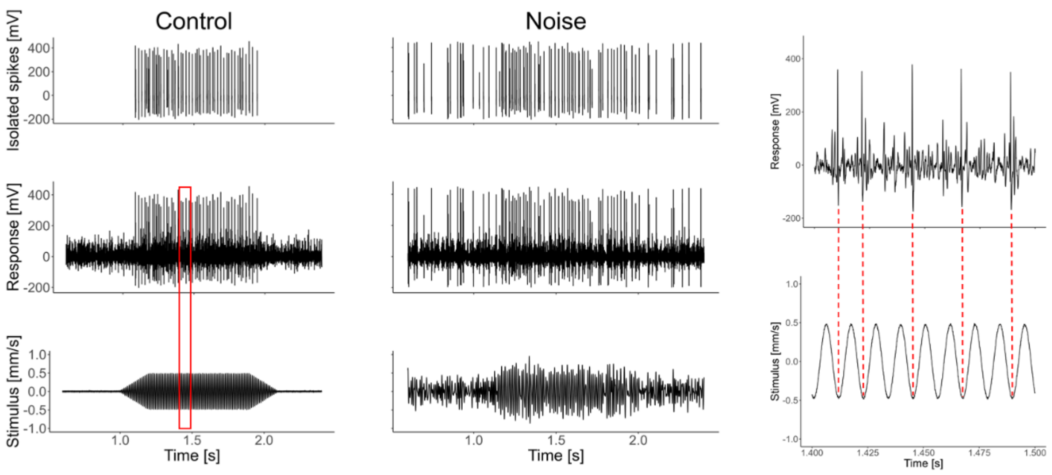

Figure S2: Left - Recording of spikes from a leg nerve of *N. viridula* male. This is an example of a recording where one can clearly see individual spikes of leg receptors that responded to FCS without noise (left) and to FCS with added 6 dB FON (right). Bottom row - stimulus; middle row - signal recorded from the nerve; top row – isolated spikes. **Right** - a closeup of the recording, where phase-locked spikes are clearly seen (area marked with a red rectangle on the left).

The time-resolved firing rate of sensory cells (i.e. PSTH) was estimated from the recordings of their summed activity. Times of individual spikes were exported to Matlab where we created an array of zeros and ones with ones positioned at the times of the spikes. The array was convolved with a Gaussian kernel with a standard deviation of 20 milliseconds. The result was a signal where each spike was substituted by the Gaussian kernel. Since the amplitude of the kernel was chosen such that the area under the kernel was one, the integral of the signal over some time interval resulted in the number of spikes in that interval. One can see that the signal corresponded to the firing rate since the integral of a firing rate over some time interval also yields the number of spikes in that interval. The width of the kernel determines the extent of

smoothing of the instantaneous firing rate. Finally, PSTH was calculated by averaging the firing rates of receptor neurons over nine repetitions of the same stimulus.

To calculate the power spectral density of the neuronal response each spike within the spike train was convolved with a Gaussian kernel. The standard deviation of the kernel was 0.5 ms. The power spectral density of the convolved spike train was calculated using the density function in R. Power spectral densities were then averaged across different treatments. To determine how much of FCS frequency was contained in the response, we measured the peak value of the averaged spectral density at 89 Hz.

Frequency threshold curve

Receptor neurons' sensitivity was tested across a wide range of frequencies from 50 to 5000 Hz, with amplitude spanning a 60 dB range in 5 dB steps. Threshold curves were expressed in acceleration units, ranging from 1.77 to 1000 cm/s^2, to facilitate comparisons with published data, where vibratory threshold is typically expressed in acceleration. Each amplitude step was tested with five consecutive 100 ms pure-tone stimuli. Spike rates were then compared to baseline activity using Student's paired T-test to determine the sensitivity threshold. Values plotted in Fig. S3 represent data from seven males, employing the same preparation and stimulation method as noise effect analysis.

SUPPLEMENTARY RESULTS

Noise impairs receptor neuron function

The threshold curve (Supplementary Fig. S3) reveals high sensitivity of the recorded neurons in the frequency range up to 2 kHz – a confirmation that in most preparations our recording captured the activity of low-, medium- and high-frequency tuned receptor neurons with tuning to frequencies below 100 Hz, around 200 Hz and 0.5-1 kHz, respectively (Čokl, 1983). The absolute threshold around 1 cm/s^2 at low frequencies (Supplementary Fig. S3) closely matches that reported previously as well, particularly for the most numerous low-frequency neurons (Čokl, 1983).

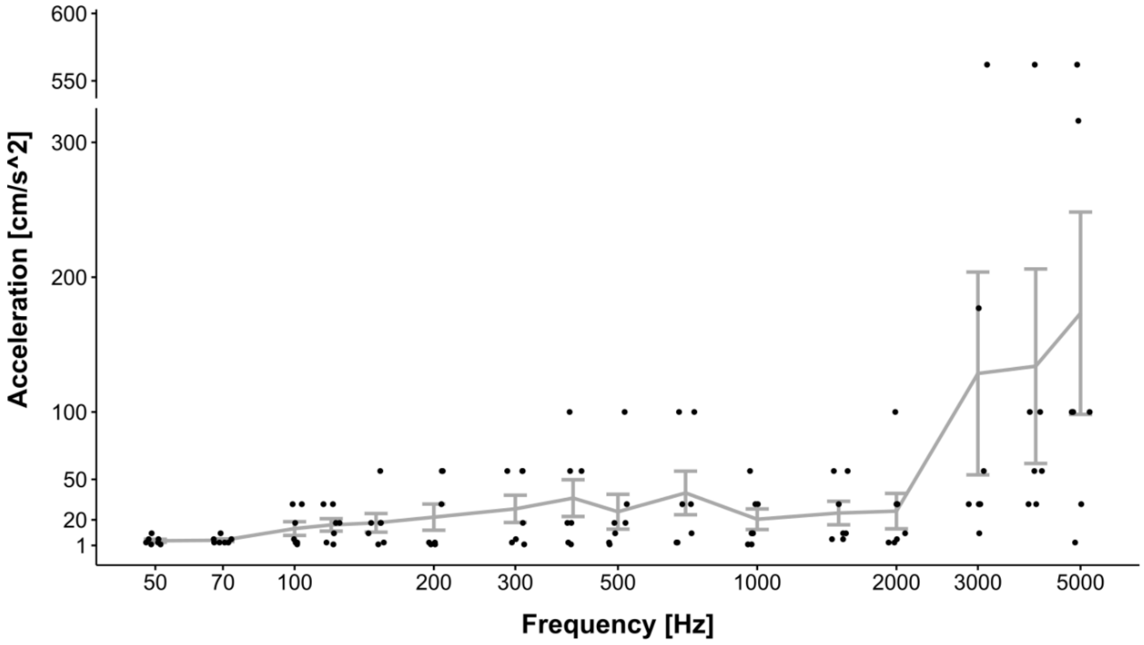

Figure S3: Threshold curve of receptor neurons (mean + SEM, n = 7), representing the lowest amplitude of pure sine wave stimuli that elicited a change in spike rate of the summed nerve activity.

Modelling the effects of noise on receptor neuron activity

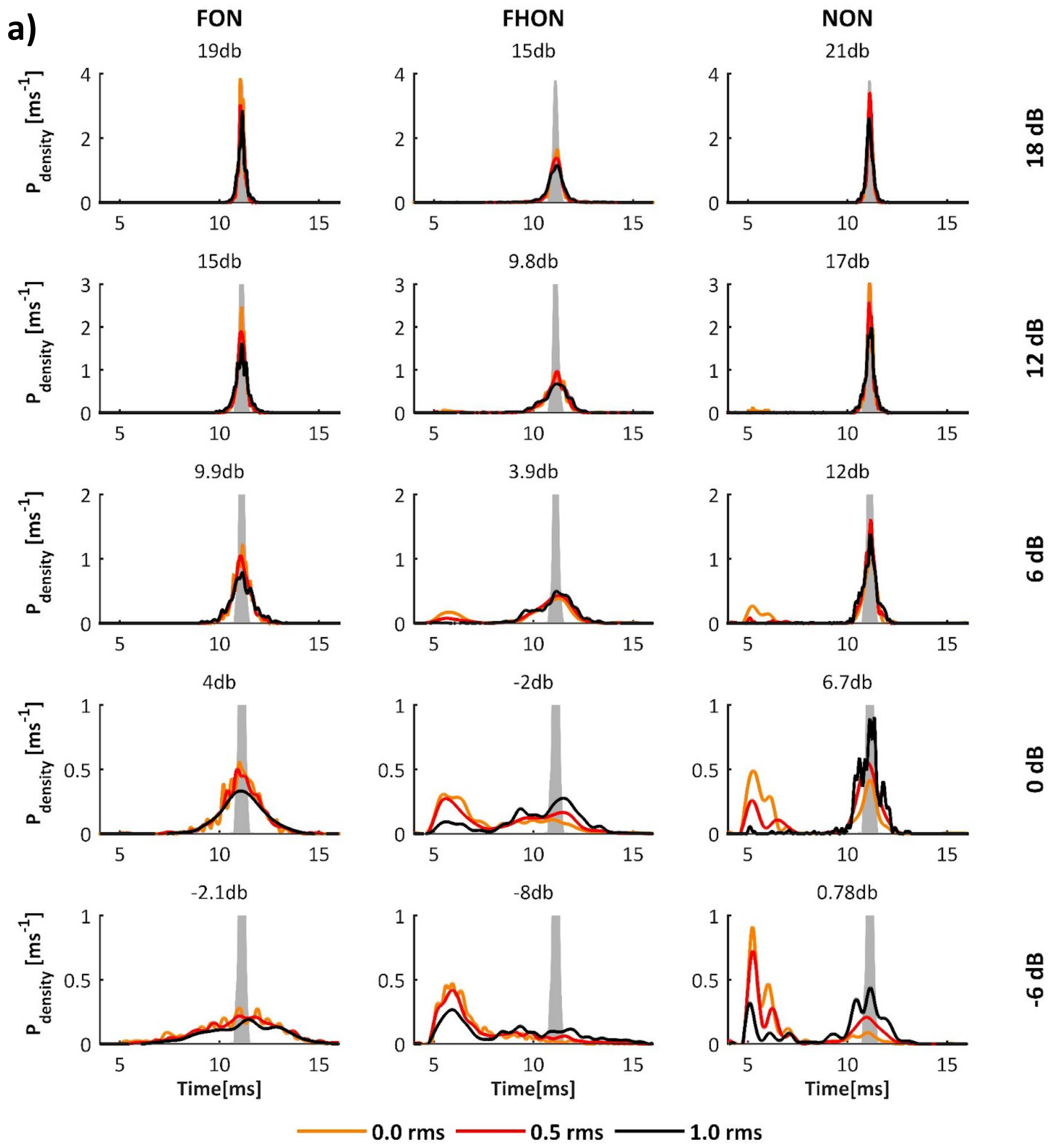

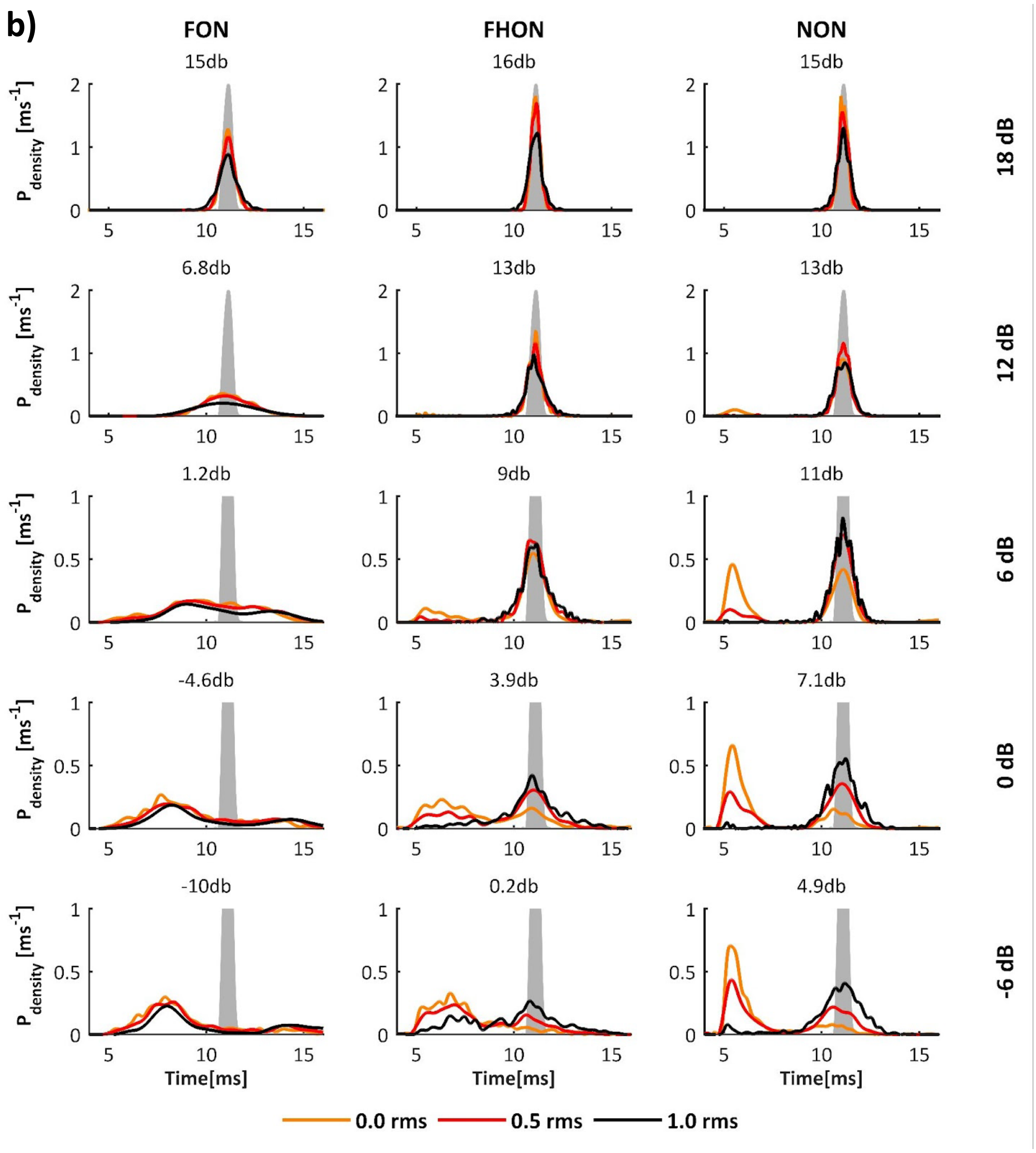

Figure S4: Further examples of distributions of intervals between vibration threshold crossings; a and b are based on recordings from two different plants (compare also with Fig. 7a where only 6 and -6 dB SNR levels for the first plant are shown). Data for FCS with a range of different SNR values and three thresholds (0.0, 0.5 and 1.0 RMS) are shown. SNR at the reference point (Fig. 2, point 7) is shown on the right in bold, while SNR measured on the ipsilateral petiole is shown above each subplot. Reference distributions for FCS without added noise and 0.0 RMS threshold are shown as grey patches (note that the upper part is not shown). Note that ranges of y-axes differ for different noise amplitudes.

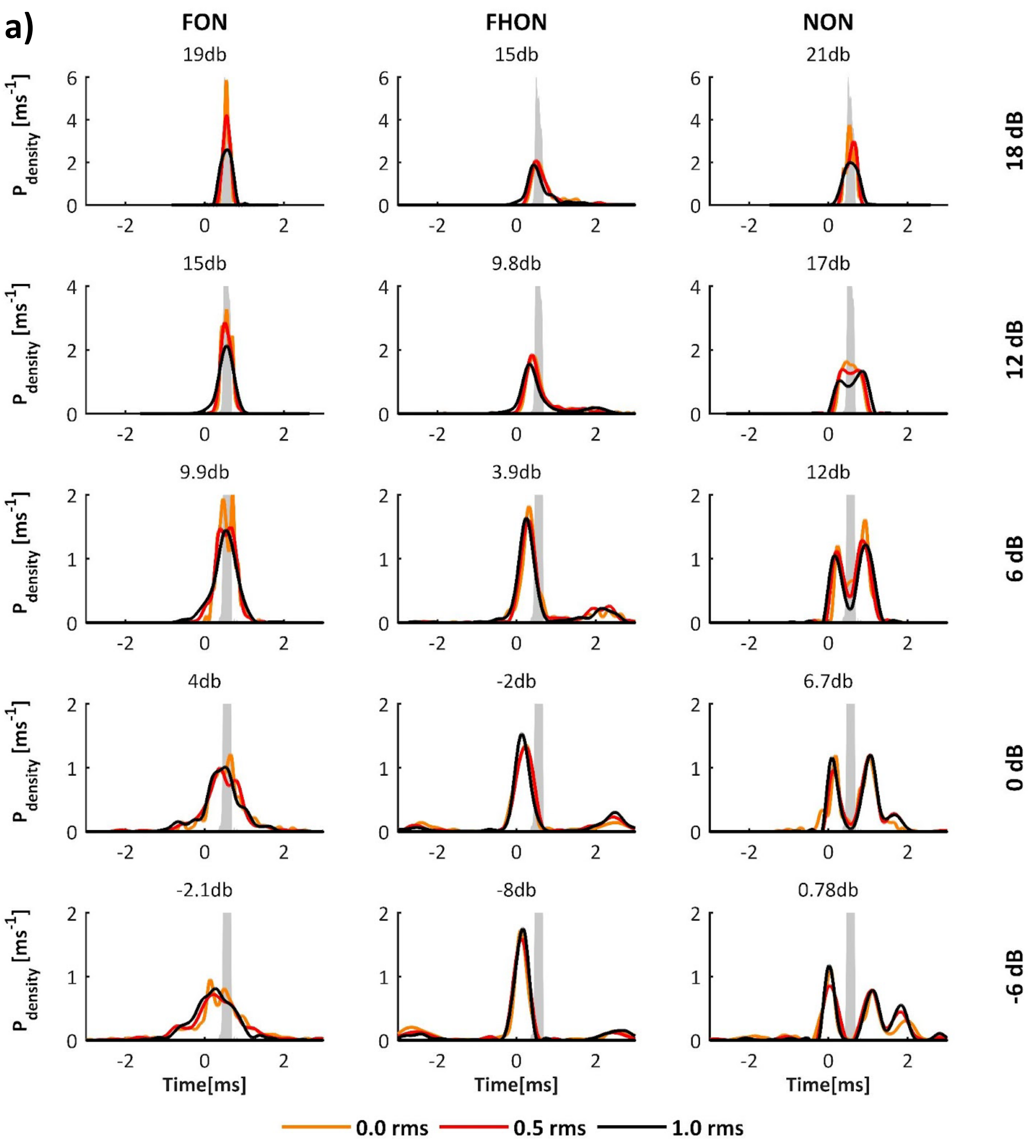

a)
FON
FHON
NON
19db
15db
21db
18 dB
15db
9.8db
17db
12 dB
9.9db
3.9db
12db
6 dB
4db
-2db
6.7db
0 dB
-2.1db
-8db
0.78db
-6 dB
P_density [ms^-1]
Time[ms]
0.0 rms
0.5 rms
1.0 rms

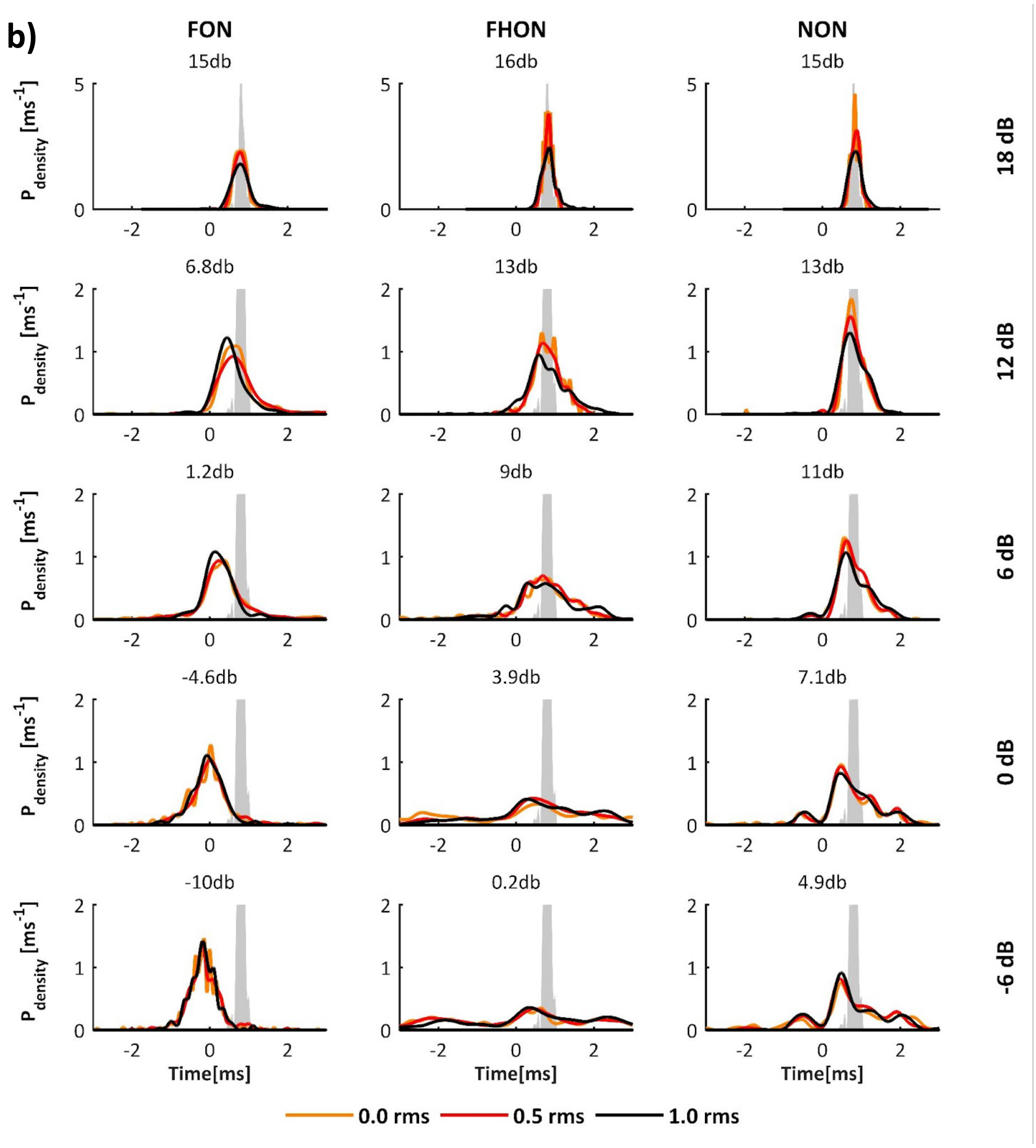

Figure S5: Further examples of distributions of delays between ipsi- and contralateral threshold crossings in a model of neurons in two spatially separated legs; a and b are based on recordings from two different plants (compare also with Fig. 7b where only 6 and -6 dB SNR for the first plant are shown). Data for FCS with a range of different SNR values and three thresholds (0.0, 0.5 and 1.0 RMS) are shown. SNR at the reference point (Fig. 2, point 7) is shown on the right in bold, while SNR measured on the ipsilateral petiole is shown above each subplot. Reference distributions for FCS without added noise and 0.0 RMS threshold are shown as grey patches (top out of scale). Positive values of the time delays indicate that the signal reached the ipsilateral point first. Note that ranges of y-axes differ for different noise amplitudes.

Table S6: Cochran's Q test

Effect tested	Chi-squared	df	p-value
Signalling animals	100.6965	9	<2.2e-12
Found vibration source	49.5354	9	1.317e-07

Table S7: Pairwise exact McNemar test

Comparison	Signalling animals	Found vibration source
Control ~ FON (6 dB)	7.84e-04	2.21e-04
Control ~ FON (0 dB)	1.72e-05	4.01e-05
Control ~ FON (-6 dB)	1.72e-05	1.91e-06
Control ~ FHON (6 dB)	2.50e-04	1.27e-02
Control ~ FHON (0 dB)	2.68e-06	4.02e-04
Control ~ FHON (-6 dB)	2.68e-06	1.53e-05
Control ~ NON (6 dB)	1.76e-01	1.22e-04
Control ~ NON (0 dB)	5.22e-02	2.75e-04
Control ~ NON (-6 dB)	2.81e-02	1.31e-03
FON (6 dB) ~ FON (0 dB)	1.38e-01	1.00e+00
FON (6 dB) ~ FON (-6 dB)	1.95e-01	4.53e-01
FON (6 dB) ~ FHON (6 dB)	8.08e-01	1.46e-01
FON (6 dB) ~ FHON (0 dB)	9.40e-03	1.00e+00
FON (6 dB) ~ FHON (-6 dB)	1.51e-02	1.00e+00
FON (6 dB) ~ NON (6 dB)	2.19e-02	5.08e-01
FON (6 dB) ~ NON (0 dB)	6.28e-02	7.54e-01
FON (6 dB) ~ NON (-6 dB)	1.58e-01	3.75e-01
FON (0 dB) ~ FON (-6 dB)	1.00e+00	6.87e-01
FON (0 dB) ~ FHON (6 dB)	2.84e-01	9.23e-02
FON (0 dB) ~ FHON (0 dB)	2.38e-01	7.54e-01
FON (0 dB) ~ FHON (-6 dB)	2.82e-01	1.00e+00
FON (0 dB) ~ NON (6 dB)	1.52e-04	2.89e-01
FON (0 dB) ~ NON (0 dB)	1.46e-03	5.49e-01
FON (0 dB) ~ NON (-6 dB)	9.49e-03	3.44e-01
FON (-6 dB) ~ FHON (6 dB)	3.25e-01	1.17e-02
FON (-6 dB) ~ FHON (0 dB)	3.58e-01	2.89e-01
FON (-6 dB) ~ FHON (-6 dB)	3.97e-01	4.53e-01
FON (-6 dB) ~ FHON (6 dB)	5.44e-04	1.09e-01
FON (-6 dB) ~ FHON (0 dB)	1.46e-03	1.25e-01
FON (-6 dB) ~ FHON (-6 dB)	5.88e-03	1.09e-01
FHON (6 dB) ~ FHON (0 dB)	3.69e-02	1.80e-01
FHON (6 dB) ~ FHON (-6 dB)	9.26e-03	1.80e-01
FHON (6 dB) ~ NON (6 dB)	2.59e-03	5.81e-01
FHON (6 dB) ~ NON (0 dB)	2.19e-02	4.81e-01

FHON (6 dB) ~ NON (-6 dB)	8.91e-02	5.08e-01
FHON (0 dB) ~ NON (-6 dB)	1.00e+00	1.00e+00
FHON (0 dB) ~ NON (6 dB)	4.29e-05	7.54e-01
FHON (0 dB) ~ NON (0 dB)	1.18e-04	1.00e+00
FHON (0 dB) ~ NON (-6 dB)	1.80e-04	7.74e-01
FHON (-6 dB) ~ NON (6 dB)	1.72e-05	5.08e-01
FHON (-6 dB) ~ NON (0 dB)	4.90e-05	6.87e-01
FHON (-6 dB) ~ NON (-6 dB)	7.65e-04	5.49e-01
NON (6 dB) ~ NON (0 dB)	7.54e-01	1.00e+00
NON (6 dB) ~ NON (-6 dB)	5.72e-01	1.00e+00
NON (0 dB) ~ NON (-6 dB)	1.00e+00	1.00e+00

Table S8: Wilcoxon signed rank-test

Comparison	Cumulative MCrS Time	Duty cycle	1st Signal Latency	Cumulative Walking
Control ~ FON (6 dB)	0.158	0.00428	0.000918	0.028
Control ~ FHON (6 dB)	0.005	0.000183	0.000183	0.004
Control ~ NON (6 dB)	0.423	0.000115	0.000183	0.058
NON (6 dB) ~ FHON (6 dB)	0.009	0.012	0.122	0.726
NON (6 dB) ~ FHON (6 dB)	0.111	0.083	0.164	0.028
FHON (6 dB) ~ NON (6 dB)	0.013	0.000458	0.001	0.289

SUPPLEMENTARY RESULTS – ADDITIONAL STATISTICAL MODELS

To confirm the initial statistical analysis, we fitted linear mixed effects models from the “lme4” package in R (Bates et al., 2015). For behavioural experiments, we fitted models for cumulative signaling, signal duty cycle, latency to the first signal and cumulative searching distance, with treatment as a fixed effect and male ID as random effect. For the proportion of males locating the FCS source, we used a binomial generalized linear mixed-effects model instead. For the proportion of signaling males, the success rate in the control group was 100% and could not be modelled this way, since the variance of this treatment is 0. For sensory physiology experiments, we fitted models for spike rate difference, spike rate during presentation of FCS in noise, spike rate during presentation of noise only and the relative power of the FCS frequency in the spike train power spectrum, with noise levels in each frequency band as a fixed effect and male ID as random effect. For all tested behavioural and neurophysiological parameters, ANOVA showed that the effect of treatment was significant. We compared estimated marginal means for treatments with the Tukey method by using the package “emmeans”. Results of post-hoc tests for secondary parameters are in the tables below. Differences from respective nonparametric tests, that were used originally in the main text, are marked with red.

Behavioural assays

Table S9: the estimated marginal means evaluated in the Tukey test based on a generalized linear mixed-effect model for cumulative signaling duration including treatment as a fixed effect and random intercepts for different male IDs. Bolded values denote significant mean differences at α = 0.05, values marked with red denote differences from the nonparametric tests used in the main text.

contrast	estimate	SE	df	t.ratio	p.value
**Control ~ FON (6 dB)**	**339.4**	**106**	**78.8**	**3.198**	**0.0105**
**Control ~ FHON (6 dB)**	**380**	**102**	**77**	**3.713**	**0.0021**
Control ~ NON (6 dB)	154.1	102	77	1.505	0.4393
FON (6 dB) ~ FHON (6 dB)	40.6	109	79.6	0.373	0.9822
FON (6 dB) ~ NON (6 dB)	-185.4	109	79.6	-1.702	0.3297
FHON (6 dB) ~ NON (6 dB)	-226	105	79	-2.142	0.1489

Table S10: the estimated marginal means evaluated in the Tukey test based on a generalized linear mixed-effect model for signal duty cycle including treatment as a fixed effect and random intercepts for different male IDs. Bolded values denote significant mean differences at α = 0.05, values marked with red denote differences from the nonparametric tests used in the main text.

contrast	estimate	SE	df	t.ratio	p.value
**Control ~ FON (6 dB)**	**0.2265**	**0.0357**	**63.6**	**6.338**	**<.0001**
**Control ~ FHON (6 dB)**	**0.3313**	**0.0374**	**64.5**	**8.853**	**<.0001**
**Control ~ NON (6 dB)**	**0.1680**	**0.0309**	**59**	**5.438**	**<.0001**
FON (6 dB) ~ FHON (6 dB)	0.1048	0.0418	63.1	2.509	0.0682
FON (6 dB) ~ NON (6 dB)	-0.0585	0.0366	62.6	-1.598	0.3874
**FHON (6 dB) ~ NON (6 dB)**	**-0.1633**	**0.0381**	**62.4**	**-4.288**	**0.0004**

Table S11: the estimated marginal means evaluated in the Tukey test based on a generalized linear mixed-effect model for latency to the first signal including treatment as a fixed effect and random intercepts for different male IDs. Bolded values denote significant mean differences at α = 0.05, values marked with red denote differences from the nonparametric tests used in the main text.

contrast	estimate	SE	df	t.ratio	p.value
**Control ~ FON (6 dB)**	**-152.67**	**48**	**65.5**	**-3.184**	**0.0117**
**Control ~ FHON (6 dB)**	**-303.68**	**50.2**	**66.9**	**-6.055**	**<.0001**
**Control ~ NON (6 dB)**	**-150.65**	**41.7**	**59.7**	**-3.614**	**0.0034**
**FON (6 dB) ~ FHON (6 dB)**	**-151.01**	**56.1**	**65.7**	**-2.692**	**0.0435**
FON (6 dB) ~ NON (6 dB)	2.02	49.2	64.7	0.041	1.000
**FHON (6 dB) ~ NON (6 dB)**	**153.03**	**51.2**	**64.6**	**2.99**	**0.0201**

Table S12: the estimated marginal means evaluated in the Tukey test based on a generalized linear mixed-effect model for cumulative searching distance including treatment as a fixed effect and random intercepts for different male IDs. Bolded values denote significant mean differences at α = 0.05, values marked with red denote differences from the nonparametric tests used in the main text.

contrast	estimate	SE	df	t.ratio	p.value
**Control ~ FON (6 dB)**	**339,4**	**106**	**78**	**3.198**	**0.0105**
**Control ~ FHON (6 dB)**	**380**	**102**	**77**	**3.713**	**0.0021**
Control ~ NON (6 dB)	154.1	102	77	1.505	0.4393
FON (6 dB) ~ FHON (6 dB)	40.6	109	79.6	0.373	0.9822
FON (6 dB) ~ NON (6 dB)	-185.4	109	79.6	-1.702	0.3297
FHON (6 dB) ~ NON (6 dB)	-226	109	79	-2.142	0.1489

Table S13: the estimated marginal means evaluated in the Tukey test based on a generalized linear mixed-effect model for percentage of males locating FCS source, including treatment as a fixed effect and random intercepts for different male IDs. Bolded values denote significant mean differences at α = 0.05, values marked with red denote differences from the nonparametric tests used in the main text. Note the infinite DF, which could imply overfitting, but since the significance results matched to the nonparametric tests and model diagnostics and convergence showed no problems, we decided to report the values here nonetheless.

contrast	estimate	SE	df	t.ratio	p.value
**Control ~ FON (6 dB)**	**2.796**	**0.670**	**Inf**	**4.173**	**0.0012**
**Control ~ FON (0 dB)**	**3.068**	**0.706**	**Inf**	**4.344**	**0.0006**
**Control ~ FON (-6 dB)**	**3.855**	**0.865**	**Inf**	**4.458**	**0.0004**
Control ~ FHON (6 dB)	1.671	0.586	Inf	2.854	0.1189
**Control ~ FHON (0 dB)**	**2.562**	**0.645**	**Inf**	**3.976**	**0.0028**
**Control ~ FHON (-6 dB)**	**2.796**	**0.670**	**Inf**	**4.173**	**0.0012**
**Control ~ NON (6 dB)**	**2.167**	**0.612**	**Inf**	**3.543**	**0.0145**
**Control ~ NON (0 dB)**	**2.356**	**0.626**	**Inf**	**3.763**	**0.0065**
**Control ~ NON (-6 dB)**	**2.167**	**0.612**	**Inf**	**3.543**	**0.0145**
FON (6 dB) ~ FON (0 dB)	0.272	0.737	Inf	0.370	1
FON (6 dB) ~ FON (-6 dB)	1.060	0.888	Inf	1.194	0.9735
FON (6 dB) ~ FHON (6 dB)	-1.124	0.635	Inf	-1.770	0.7545
FON (6 dB) ~ FHON (0 dB)	-0.233	0.682	Inf	-0.342	1
FON (6 dB) ~ FHON (-6 dB)	0.000	0.704	Inf	0.000	1
FON (6 dB) ~ NON (6 dB)	-0.628	0.654	Inf	-0.960	0.9943
FON (6 dB) ~ NON (0 dB)	-0.440	0.666	Inf	-0.661	0.9997
FON (6 dB) ~ NON (-6 dB)	-0.628	0.654	Inf	-0.960	0.9943
FON (0 dB) ~ FON (-6 dB)	0.787	0.914	Inf	0.861	0.9975
FON (0 dB) ~ FHON (6 dB)	-1.397	0.672	Inf	-2.077	0.5438
FON (0 dB) ~ FHON (0 dB)	-0.506	0.716	Inf	-0.706	0.9995
FON (0 dB) ~ FHON (-6 dB)	-0.272	0.737	Inf	-0.370	1
FON (0 dB) ~ NON (6 dB)	-0.901	0.690	Inf	-1.305	0.9527
FON (0 dB) ~ NON (0 dB)	-0.713	0.701	Inf	-1.017	0.9914
FON (0 dB) ~ NON (-6 dB)	-0.901	0.690	Inf	-1.305	0.9527
FON (-6 dB) ~ FHON (6 dB)	-2.184	0.836	Inf	-2.614	0.2109
FON (-6 dB) ~ FHON (0 dB)	-1.293	0.870	Inf	-1.486	0.8981
FON (-6 dB) ~ FHON (-6 dB)	-1.060	0.888	Inf	-1.194	0.9735

FON (-6 dB) ~ FHON (6 dB)	-1.688	0.849	Inf	-1.987	0.6079
FON (-6 dB) ~ FHON (0 dB)	-1.500	0.858	Inf	-1.748	0.7677
FON (-6 dB) ~ FHON (-6 dB)	-1.688	0.849	Inf	-1.987	0.6079
FHON (6 dB) ~ FHON (0 dB)	0.891	0.610	Inf	1.461	0.9072
FHON (6 dB) ~ FHON (-6 dB)	1.124	0.635	Inf	1.770	0.7545
FHON (6 dB) ~ NON (6 dB)	0.496	0.577	Inf	0.859	0.9976
FHON (6 dB) ~ NON (0 dB)	0.684	0.591	Inf	1.157	0.9785
FHON (6 dB) ~ NON (-6 dB)	0.496	0.577	Inf	0.859	0.9976
FHON (0 dB) ~ NON (-6 dB)	0.233	0.682	Inf	0.342	1
FHON (0 dB) ~ NON (6 dB)	-0.395	0.630	Inf	-0.627	0.9998
FHON (0 dB) ~ NON (0 dB)	-0.207	0.642	Inf	-0.322	1
FHON (0 dB) ~ NON (-6 dB)	-0.395	0.630	Inf	-0.627	0.9998
FHON (-6 dB) ~ NON (6 dB)	-0.628	0.654	Inf	-0.960	0.9943
FHON (-6 dB) ~ NON (0 dB)	-0.440	0.666	Inf	-0.661	0.9997
FHON (-6 dB) ~ NON (-6 dB)	-0.628	0.654	Inf	-0.960	0.9943
NON (6 dB) ~ NON (0 dB)	0.188	0.612	Inf	0.307	1
NON (6 dB) ~ NON (-6 dB)	0.000	0.599	Inf	0.000	1
NON (0 dB) ~ NON (-6 dB)	-0.188	0.612	Inf	-0.307	1

Sensory physiology

Table S14: the estimated marginal means evaluated in the Tukey test based on a linear mixed-effect model for **the spike rate difference**, including different noise levels within a noise frequency band as a fixed effect and random intercepts for different male IDs. Bolded values denote significant mean differences at α = 0.05, values marked with red denote differences from the nonparametric tests used in the main text.

contrast	estimate	SE	df	t.ratio	p.value
Control ~ FON (24 dB)	1.38	3.93	55	0.351	0.9985
Control ~ FON (18 dB)	7.57	3.93	55	1.927	0.2629
**Control ~ FON (12 dB)**	**17.41**	**3.93**	**55**	**4.429**	**0.0002**
**Control ~ FON (6 dB)**	**34.98**	**3.93**	**55**	**8.9**	**<.0001**
**Control ~ FON (0 dB)**	**49.47**	**3.93**	**55**	**12.587**	**<.0001**
Control ~ FHON (24 dB)	0.972	3.08	55	0.316	0.9991
Control ~ FHON (18 dB)	2.627	3.08	55	0.854	0.9203
Control ~ FHON (12 dB)	8.107	3.08	55	2.634	0.0535
**Control ~ FHON (6 dB)**	**16.883**	**3.08**	**55**	**5.485**	**<.0001**
**Control ~ FHON (0 dB)**	**31.953**	**3.08**	**55**	**10.381**	**<.0001**
Control ~ NON (24 dB)	-0.834	2.53	55	-0.33	0.9989
Control ~ NON (18 dB)	-0.541	2.53	55	-0.214	0.9999
Control ~ NON (12 dB)	-0.216	2.53	55	-0.085	1
Control ~ NON (6 dB)	1.211	2.53	55	0.479	0.9934
Control ~ NON (0 dB)	3.222	2.53	55	1.275	0.688

Table S15: the estimated marginal means evaluated in the Tukey test based on a linear mixed-effect model for **the spike rate during presentation of FCS in noise**, including different noise levels within a noise frequency band as a fixed effect and random intercepts for different male IDs. Bolded values denote significant mean differences at α = 0.05, values marked with red denote differences from the nonparametric tests used in the main text.

contrast	estimate	SE	df	t.ratio	p.value
Control ~ FON (24 dB)	-0.171	3.91	55	-0.044	1
Control ~ FON (18 dB)	2.76	3.91	55	0.706	0.9631
Control ~ FON (12 dB)	5.162	3.91	55	1.321	0.6558
Control ~ FON (6 dB)	8.916	3.91	55	2.281	0.1254
**Control ~ FON (0 dB)**	**13.269**	**3.91**	**55**	**3.395**	**0.0064**
Control ~ FHON (24 dB)	1.24	2.78	55	0.445	0.9953

Control ~ FHON (18 dB)	1.59	2.78	55	0.571	0.9853
Control ~ FHON (12 dB)	2.18	2.78	55	0.785	0.9428
Control ~ FHON (6 dB)	1.67	2.78	55	0.603	0.9813
Control ~ FHON (0 dB)	3.06	2.78	55	1.102	0.7999
Control ~ NON (24 dB)	-0.334	2.45	55	-0.136	1
Control ~ NON (18 dB)	-1.623	2.45	55	-0.661	0.9721
Control ~ NON (12 dB)	-4.806	2.45	55	-1.958	0.2477
**Control ~ NON (6 dB)**	**-7.089**	**2.45**	**55**	**-2.888**	**0.0274**
**Control ~ NON (0 dB)**	**-7.718**	**2.45**	**55**	**-3.144**	**0.0134**

Table S16: the estimated marginal means evaluated in the Tukey test based on a linear mixed-effect model **for the spike rate during presentation of noise only**, including different noise levels within a noise frequency band as a fixed effect and random intercepts for different male IDs. Bolded values denote significant mean differences at α = 0.05, values marked with red denote differences from the nonparametric tests used in the main text.

contrast	estimate	SE	df	t.ratio	p.value
Control ~ FON (24 dB)	-1.55	3.59	55	-0.433	0.9959
Control ~ FON (18 dB)	-4.81	3.59	55	-1.342	0.6407
**Control ~ FON (12 dB)**	**-12.24**	**3.59**	**55**	**-3.414**	**0.006**
**Control ~ FON (6 dB)**	**-26.07**	**3.59**	**55**	**-7.269**	**<.0001**
**Control ~ FON (0 dB)**	**-36.21**	**3.59**	**55**	**-10.096**	**<.0001**
Control ~ FHON (24 dB)	0.264	1.85	55	0.143	1
Control ~ FHON (18 dB)	-1.041	1.85	55	-0.563	0.9862
**Control ~ FHON (12 dB)**	**-5.927**	**1.85**	**55**	**-3.207**	**0.0111**
**Control ~ FHON (6 dB)**	**-15.209**	**1.85**	**55**	**-8.229**	**<.0001**
**Control ~ FHON (0 dB)**	**-28.892**	**1.85**	**55**	**-15.633**	**<.0001**
Control ~ NON (24 dB)	0.5	1.48	55	0.338	0.9987
Control ~ NON (18 dB)	-1.08	1.48	55	-0.732	0.957
**Control ~ NON (12 dB)**	**-4.59**	**1.48**	**55**	**-3.107**	**0.0149**
**Control ~ NON (6 dB)**	**-8.3**	**1.48**	**55**	**-5.617**	**<.0001**
**Control ~ NON (0 dB)**	**-10.94**	**1.48**	**55**	**-7.404**	**<.0001**

Table S17: the estimated marginal means evaluated in the Tukey test based on a linear mixed-effect model **for the relative power of the FCS frequency in the spike train power spectrum**, including different noise levels within a noise frequency band as a fixed effect and random intercepts for different male IDs. Bolded values denote significant mean differences at α = 0.05, values marked with red denote differences from the nonparametric tests used in the main text.

contrast	estimate	SE	df	t.ratio	p.value
FON (24 dB) ~ FON (18 dB)	0.0997	0.0941	55	1.06	0.7515
**FON (24 dB) ~ FON (12 dB)**	**0.415**	**0.0941**	**55**	**4.413**	**0.0002**
**FON (24 dB) ~ FON (6 dB)**	**0.6355**	**0.0941**	**55**	**6.756**	**<.0001**
**FON (24 dB) ~ FON (0 dB)**	**0.6745**	**0.0941**	**55**	**7.172**	**<.0001**
FHON (24 dB) ~ FHON (18 dB)	0.0334	0.0774	55	0.432	0.9878
**FHON (24 dB) ~ FHON (12 dB)**	**0.2169**	**0.0774**	**55**	**2.804**	**0.0276**
**FHON (24 dB) ~ FHON (6 dB)**	**0.5111**	**0.0774**	**55**	**6.606**	**<.0001**
**FHON (24 dB) ~ FHON (0 dB)**	**0.7173**	**0.0774**	**55**	**9.272**	**<.0001**
NON (24 dB) ~ NON (18 dB)	-0.0746	0.148	55	-0.506	0.9781
NON (24 dB) ~ NON (12 dB)	0.0381	0.148	55	0.258	0.9983
NON (24 dB) ~ NON (6 dB)	0.2185	0.148	55	1.48	0.4643
**NON (24 dB) ~ NON (0 dB)**	**0.4459**	**0.148**	**55**	**3.022**	**0.0152**

REFERENCES

- Brumm, H., and Slabbekoorn, H. (2005). Acoustic communication in noise. Adv. Stud. Behav. 35, 151-209.
- Classen-Rodríguez, L., Tinghitella, R., and Fowler-Finn, K. (2021). Anthropogenic noise affects insect and arachnid behavior, thus changing interactions within and between species. Curr. Opin. Insect Sci. 47, 142-153.
- Čokl, A. (2000). Stink bug interaction with host plants during communication. J. Insect Physiol. 54, 1113-11245.
- Hill, P.S.M., and Wessel, A. (2016). Biotremology. Curr. Biol. 26, R187–R191.
- Howard, D.M., and Angus, J.A.S. (2009). Acoustics and psychoacoustics, 4th ed. Focal Press, Oxford.
- Kidd, G. Jr., Mason, C. R., Richards, V. M., Gallun, F. J., and Durlach, N. I. (2008). Informational masking. In: Yost, W. A., Popper, M. A., Fay, R. R., Auditory Perception of Sound Sources. Springer Handbook of Auditory Research vol 29, pp. 143–189. Springer, New York
- Raboin, M., and Elias, D.O. (2019). Anthropogenic noise and the bioacoustics of terrestrial invertebrates. J. Exp. Biol. 222, jeb178749.
- Strauß, J., Stritih-Peljhan, N., Lakes-Harlan, R., 2019. Determining vibroreceptor sensitivity in insects: the influence of experimental parameters and recording techniques. In: Hill, P.S.M., Lakes-Harlan, R., Mazzoni, V., Narins, P.M., Virant-Doberlet, M., Wessel, A. (Eds.), Biotremology: Studying Vibrational Behavior. Springer, Cham, pp. 209–233.
- Šturm, R., Polajnar, J., and Virant-Doberlet, M. (2019). Practical issues in studying natural vibroscape and biotic noise. In Biotremology – Studying Vibrational Behavior, P.S.M. Hill, R. Lakes-Harlan, V. Mazzoni, P.M. Narins, M. Virant-Doberlet, and A. Wessel, eds. (Springer), pp. 125-148.
- Yost, W.A. (2008). Perceiving sound sources. In: Yost, W.A., Popper, A.N., Fay, R.R. (Eds) Auditory perception of sound sources. Springer handbook of auditory research vol 29, pp. 1–12. Springer, New York.
