# Supplementary material for: Vibrational noise disrupts Nezara viridula communication, irrespective of spectral overlap
Source: Commun Biol. 2024 Nov 19;7:1533. doi: 10.1038/s42003-024-07185-3 (PMC11577028; doi:10.1038/s42003-024-07185-3)
Supplement: Supplementary file 3 — Reporting Summary [file 42003_2024_7185_MOESM3_ESM.pdf]

nature portfolio

Corresponding author(s): Jernej Polajnar

Last updated by author(s): Oct 15, 2024

Reporting Summary

Nature Portfolio wishes to improve the reproducibility of the work that we publish. This form provides structure for consistency and transparency in reporting. For further information on Nature Portfolio policies, see our Editorial Policies and the Editorial Policy Checklist.

Statistics

For all statistical analyses, confirm that the following items are present in the figure legend, table legend, main text, or Methods section.

n/a	Confirmed	
☐	☒	The exact sample size (*n*) for each experimental group/condition, given as a discrete number and unit of measurement
☐	☒	A statement on whether measurements were taken from distinct samples or whether the same sample was measured repeatedly
☐	☒	The statistical test(s) used AND whether they are one- or two-sided<br>*Only common tests should be described solely by name; describe more complex techniques in the Methods section.*
☒	☐	A description of all covariates tested
☐	☒	A description of any assumptions or corrections, such as tests of normality and adjustment for multiple comparisons
☐	☒	A full description of the statistical parameters including central tendency (e.g. means) or other basic estimates (e.g. regression coefficient) AND variation (e.g. standard deviation) or associated estimates of uncertainty (e.g. confidence intervals)
☐	☒	For null hypothesis testing, the test statistic (e.g. F, t, r) with confidence intervals, effect sizes, degrees of freedom and P value noted<br>*Give P values as exact values whenever suitable.*
☒	☐	For Bayesian analysis, information on the choice of priors and Markov chain Monte Carlo settings
☒	☐	For hierarchical and complex designs, identification of the appropriate level for tests and full reporting of outcomes
☒	☐	Estimates of effect sizes (e.g. Cohen's *d*, Pearson's *r*), indicating how they were calculated

Our web collection on statistics for biologists contains articles on many of the points above.

Software and code

Policy information about availability of computer code

Data collection	Commercial software used for data collection is listed in the manuscript and the supplement.
Data analysis	Commercial software used for data analysis is listed in the manuscript and the supplement, while custom code will be published in the accompanying dataset (see the section "Data" below).

For manuscripts utilizing custom algorithms or software that are central to the research but not yet described in published literature, software must be made available to editors and reviewers. We strongly encourage code deposition in a community repository (e.g. GitHub). See the Nature Portfolio guidelines for submitting code & software for further information.

Data

Policy information about availability of data

All manuscripts must include a data availability statement. This statement should provide the following information, where applicable:

- Accession codes, unique identifiers, or web links for publicly available datasets
- A description of any restrictions on data availability
- For clinical datasets or third party data, please ensure that the statement adheres to our policy

All information needed to reproduce the results of the paper is in the paper and the Supplementary Materials. Raw data extracted from audio and video recordings and electrophysiological recordings are archived at the Zenodo data repository (Janža et al. 2024; DOI: 10.5281/zenodo.11910334). Due to the large file sizes, original audio and video recordings are available per reasonable request to the authors.

Research involving human participants, their data, or biological material

Policy information about studies with human participants or human data. See also policy information about sex, gender (identity/presentation), and sexual orientation and race, ethnicity and racism.

Reporting on sex and gender	N/A
Reporting on race, ethnicity, or other socially relevant groupings	N/A
Population characteristics	N/A
Recruitment	N/A
Ethics oversight	N/A

Note that full information on the approval of the study protocol must also be provided in the manuscript.

Field-specific reporting

Please select the one below that is the best fit for your research. If you are not sure, read the appropriate sections before making your selection.

- [x] Life sciences
- [ ] Behavioural & social sciences
- [ ] Ecological, evolutionary & environmental sciences

For a reference copy of the document with all sections, see nature.com/documents/nr-reporting-summary-flat.pdf

Life sciences study design

All studies must disclose on these points even when the disclosure is negative.

Sample size	Sample size for behavioural experiments was limited by the availability and activity of experimental animals. We managed to obtain sufficient number of males active in all trials for testing the effect of noise on continuous variables for one SNR level only, while N=30 sufficed for comparisons of searching/signalling activity (binomial) across all treatments.
Data exclusions	No data were excluded.
Replication	Models of intervals and delays between vibration threshold crossings built from measurements on a single plant were verified by taking measurements from a second plant (see Supplemental results).
Randomization	Males were allocated to treatment groups in behavioural experiments by haphazardly picking them from the collection boxes. During this process, variability between the animals was not readily apparent.
Blinding	Blinding during the experiments was not possible because the investigators had to monitor vibrational emissions in real time, thus also seeing the stimulation. No treatment group-specific analysis was performed, therefore blinding in the data analysis step was not necessary.

Reporting for specific materials, systems and methods

We require information from authors about some types of materials, experimental systems and methods used in many studies. Here, indicate whether each material, system or method listed is relevant to your study. If you are not sure if a list item applies to your research, read the appropriate section before selecting a response.

Materials & experimental systems

n/a	Involved in the study	
☒	☐	Antibodies
☒	☐	Eukaryotic cell lines
☒	☐	Palaeontology and archaeology
☐	☒	Animals and other organisms
☒	☐	Clinical data
☒	☐	Dual use research of concern
☒	☐	Plants

Methods

n/a	Involved in the study	
☒	☐	ChIP-seq
☒	☐	Flow cytometry
☒	☐	MRI-based neuroimaging

Animals and other research organisms

Policy information about studies involving animals; ARRIVE guidelines recommended for reporting animal research, and Sex and Gender in Research

Laboratory animals	The study did not involve laboratory animals.
Wild animals	Adult Nezara viridula bugs were collected in autumn in the coastal part of Slovenia by beat sampling and transported to the premises of the National Institute of Biology by car in plastic boxes along with cuttings of the host plants (Clematis vitalba).<br><br>After the experiments, the animals were kept in the institutes' rearing room until natural death.
Reporting on sex	Findings apply to male Nezara viridula only. The animals were sexed on the basis of external morphology.
Field-collected samples	The study did not involve samples collected from the field.
Ethics oversight	No ethical approval or guidance was required - the study was done on insects (i.e., not vertebrates or higher invertebrates), and the species is considered invasive in the area where specimens were collected, thus it is not protected.

Note that full information on the approval of the study protocol must also be provided in the manuscript.

Plants

Seed stocks	*Report on the source of all seed stocks or other plant material used. If applicable, state the seed stock centre and catalogue number. If plant specimens were collected from the field, describe the collection location, date and sampling procedures.*
Novel plant genotypes	*Describe the methods by which all novel plant genotypes were produced. This includes those generated by transgenic approaches, gene editing, chemical/radiation-based mutagenesis and hybridization. For transgenic lines, describe the transformation method, the number of independent lines analyzed and the generation upon which experiments were performed. For gene-edited lines, describe the editor used, the endogenous sequence targeted for editing, the targeting guide RNA sequence (if applicable) and how the editor was applied.*
Authentication	*Describe any authentication procedures for each seed stock used or novel genotype generated. Describe any experiments used to assess the effect of a mutation and, where applicable, how potential secondary effects (e.g. second site T-DNA insertions, mosiacism, off-target gene editing) were examined.*
